# Medication-related burden among Iraqi patients with ulcerative colitis: a cross-sectional study

**DOI:** 10.25122/jml-2023-0342

**Published:** 2024-08

**Authors:** Nawar Abdulridha Abood, Dheyaa Jabbar Kadhim, Raghad Jawad Hussein

**Affiliations:** 1 Department of Clinical Pharmacy, College of Pharmacy, University of Baghdad, Baghdad, Iraq; 2 The Gastroenterology and Hepatology Teaching Hospital, Baghdad Medical City, Baghdad, Iraq

**Keywords:** Inflammatory bowel disease, Iraq, Living with Medicines Questionnaire, medication-related burden, ulcerative colitis, CD, Crohn's Disease, FMT, Fecal Microbiota Transplantation, HPCs, Healthcare Professionals, IBD, Inflammatory Bowel Disease, IQR, Interquartile Range, ID, Iraqi Dinar, LMQ, Living with Medicines Questionnaire, MRB, Medication-Related Burden, MOH, Ministry of Health, QoL, Quality of Life, r, Regression Coefficient, SD, Standard Deviation, UC, Ulcerative Colitis

## Abstract

Ulcerative colitis (UC) is a chronic inflammatory bowel disease characterized by recurring periods of inflammation and remission, primarily affecting the colon. The concept of medication-related burden, which refers to the adverse effects experienced by patients due to conventional medical treatments, is relatively new in the field. This study aimed to measure medication-related burden among patients with ulcerative colitis in Iraq. The study was conducted at the Gastroenterology and Hepatology Teaching Hospital, Medical City, Baghdad, Iraq, from December 2022 to May 2023. We used the Arabic version of the Living with Medicines Questionnaire version 3 (LMQ-3) to explore medication-related burdens experienced by patients with UC. Eighty-six patients with ulcerative colitis were included. The mean of the total medication-related burden score was 107.5 ± 20.7. The findings showed that 45.3% of patients with UC had a moderate degree of medication-related burden, followed by minimum burden (44.2%), high burden (5.8%), and no burden (4.7%). The lowest median burden scores emerged in five domains: interactions with healthcare professionals, practical difficulties with medication use, medication side effects, medication effectiveness, and the impact on daily life. Conversely, the highest-burden scores were noted in the cost, concerns about medication use, and autonomy to vary the regimen domains. In multivariate analysis, none of the patient-related variables was independently correlated with the total medication-related burden score. A large proportion of the patients with UC who participated in the current study reported varying degrees of medication-related burden, with the majority having a minimum to moderate medication-related burden.

## INTRODUCTION

Inflammatory bowel disease (IBD) is a chronic condition characterized by recurrent and unexplained inflammation of the gastrointestinal tract. It encompasses two main subtypes, namely Crohn's disease (CD) and ulcerative colitis (UC) [1,2]. UC specifically affects the colon and is characterized by recurrent inflammation [3]. The etiology of UC is multifactorial; previous studies suggest that genetic factors significantly contribute to IBD development among the Iraqi Arab population [4-6]. In addition, diet plays an important role in the development of IBD or the protection against it [7]. There is a growing incidence of IBD in the Arab world [8], with reported UC incidence rates ranging from 1.35/100,000 in Oman to 4.98/100,000 in Iran and prevalence rates ranging from 35.52/100,000 in Iran to 106.2/100,000 in Lebanon [4]. The clinical features of IBD depend on the site of the disease as well as the severity of the inflammation. Gastrointestinal symptoms include abdominal pain, rectal bleeding, diarrhea, bloody mucus, and rectal urgency [9]. Extraintestinal involvement of IBD, which can affect joints, eyes, and skin, occurs in 25% of patients [3].

Treatment of IBD typically involves a combination of anti-inflammatory, immunosuppressive, and immunomodulatory drugs aimed at controlling disease activity. Despite advances, a definitive cure for ulcerative colitis has not been established. The goals of current therapies are to achieve remission of the active disease and sustain this remission for an extended duration, all while preserving a high quality of life (QoL) and reducing the necessity for surgical intervention [10]. The pharmacological treatment for IBD encompasses a range of drugs, such as salicylates, corticosteroids, calcineurin inhibitors, thiopurines, biologics, and Janus kinase inhibitors. Therapeutic interventions can be categorized into induction therapy and maintenance therapy. Furthermore, the implementation of treatment techniques is frequently influenced by the severity and scope of the disease [11]. Fecal microbiota transplantation (FMT) is a novel therapeutic approach for UC. Nevertheless, the use of FMT in clinical practice is controversial, mostly due to the lack of conclusive evidence about its efficacy [12].

The combination of advancements in pharmaceutical research and development, effective public health measures, and improvements in overall living conditions have played a crucial role in achieving a substantial decline in death rates and a notable increase in life expectancy. Nevertheless, in the context of increased lifespan, many patients experience the coexistence of numerous medical conditions and the need for different pharmacological treatments. The judicious utilization of medications can effectively improve patients' treatment outcomes. Nevertheless, the prolonged use of medications typically leads to adverse repercussions. Potential consequences may encompass adverse medication events, the impact on daily functioning, and the effects on many aspects of well-being, such as social, financial, psychological, and functional domains [13]. Aggregating these difficulties has directly contributed to the emergence of the 'treatment burden'. Despite the absence of a precise and universally accepted definition for treatment burden, some scholars have argued that the treatment regimens impose a considerable 'workload'. [14]. Medication-related burden (MRB) is a big component of the treatment burden [15]. Individuals diagnosed with various chronic health illnesses are especially prone to experiencing a sense of burden associated with their treatment regimen because they are frequently required to partake in a multifaceted sequence of self-care tasks to maintain their overall well-being [16]. Patients frequently encounter MRB because of the established patterns linked to medication intake, unfavorable effects, the intricacy of treatment regimens, difficulties associated with the healthcare system (such as limited access to medications), and disruptions to their social engagements. The presence of MRB can adversely affect individuals' social, psychological, and physical well-being [17].

It is crucial to evaluate the patient's perspective on the medication regimen and identify potential obstacles that could hinder optimal medication utilization. In the context of Iraq, previous research has examined the prevalence of MRB among patients with various chronic illnesses [18-20]. However, to the best of our knowledge, no prior study has specifically investigated MRB among patients with UC in Iraq. The objective of the present study was to assess MRB in Iraqi patients with UC and investigate potential associations between sociodemographic and disease-related factors and the extent of MRB reported by patients with UC.

## MATERIAL AND METHODS

### Study design and population

A cross-sectional study was conducted at the Gastroenterology and Hepatology Teaching Hospital, Medical City, Baghdad, Iraq, from December 2022 to May 2023. The inclusion criteria were patients with UC aged ≥18 years, diagnosed with UC at least 6 months before the study, who regularly used at least one medication for UC. The exclusion criteria were patients with cognitive, hearing, or speech deficits, pregnancy, and patients who did not consent to participate.

### Study questionnaire

The questionnaire was structured in two sections. The first section contained questions related to patients’ sociodemographic and clinical information. The second section included the Arabic version of the Living with Medicines Questionnaire version 3 (LMQ-3) [21] to evaluate MRB. The LMQ-3 consists of 41 items distributed across eight domains: relationships with healthcare professionals, practical difficulties in medicine use, cost-related burden, side effects, medication effectiveness, concerns about medicine use, the impact on daily life, and autonomy in regimen adjustment. Responses were recorded on a five-point Likert scale. The overall MRB was quantified by summing the scores of all domains, with possible scores ranging from 41 to 205; lower scores suggest a lesser MRB [22].

### Questionnaire administration

The data collection for this investigation was conducted only by the researcher. After explaining the purpose of the study to participants, the investigator recorded patient responses to the LMQ-3, which typically took between 20 to 30 minutes to complete.

### Statistical analysis

The normality of continuous variables was assessed using the Anderson-Darling test. Variables with normal distribution were described using the mean and standard deviation (SD). In contrast, variables that did not have a normal distribution were described using the median and interquartile range (IQR). Linear regression analysis evaluated the association between LMQ and study predictors. A multivariate linear regression with backward elimination was employed for more complex analyses, removing variables with a probability of F ≥ 0.10. The study was conducted using SPSS 27.1 software. A *P* value less than 0.05 (two-tailed) was considered statistically significant.

## RESULTS

In the present study, 86 patients with UC were included. Their sociodemographic, clinical, and disease characteristics are presented in Table 1.

**Table 1:** Sociodemographic, clinical, and disease characteristics of patients with UC

Variable		Mean ± SD	Variable		Mean ± SD
Age (y) mean ± SD		37.8 ± 12.8	Disease duration (y)		9.6 ± 6.5
		Number (%)			Number (%)
Sex	Female	45 (52.3%)	Source of medication	MOH	27 (31.4%)
	Male	41 (47.7%)		Private and MOH	59 (68.6%)
Social	Single	18 (20.9%)	Biological type	Original	25 (29.1%)
	Married	68 (79.1%)		Biosimilar	61 (70.9%)
Education	Illiterate	4 (4.7%)	No. of co-existing disease(s)	No other disease	68 (79.1%)
	Primary	19 (22.1%)		Single	14 (16.3%)
	Secondary	25 (29.1%)		Two	4 (4.7%)
	College	38 (44.2%)	No. chronic medications	1	15 (17.44)
Place of residence	Urban	69 (80.2%)		2	43 (50%)
	Rural	17 (19.8%)		3	18 (20.93)
Governorate	Baghdad	46 (53.5%)		≥4	10 (11.63%)
	Others	40 (46.5%)			
Smoking	Yes	6 (7.0%)			
	No	79 (93.0%)			
Income	Low (<0.5 million ID)	36 (41.9%)			
	Intermediate (0.5–1.0 million ID)	40 (46.5%)			
	High (>1.0 million ID)	10 (11.6%)			

ID, Iraqi dinar; MOH, Ministry of Health; No, number.

**Figure 1:**
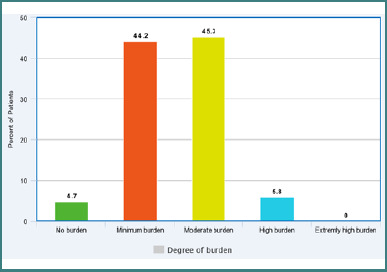
Level of MRB according to the total LMQ score

The mean of the total LMQ score was 107.5 ± 20.7. The findings showed that 45.3% of the patients with UC had a moderate degree of MRB, followed by minimum burden (44.2%), high burden (5.8%), and no burden (4.7%) (Table 2 and Figure 1).

**Table 2:** Perceived level of medication burden

Total LMQ score (mean ± SD)	107.5 ± 20.7	
Degree of burden	The range of each category	Number (%)
No burden	41-73	4 (4.7%)
Minimum burden	74-106	38 (44.2%)
Moderate burden	107-139	39 (45.3%)
High burden	140-172	5 (5.8%)
Extremely high burden	173–205	0.0 (0.0%)

LMQ, Living with Medicines Questionnaire.

The results showed that five domains of the LMQ-3 for patients with UC had lower median burden scores, indicating a lesser medication-related burden. These domains included relationships with healthcare professionals, practical difficulties in using medicines, side effects, effectiveness of prescribed medications, and the impact of using medicines on daily life. On the other hand, the domains of cost-related burden, concerns about medicines use, and autonomy to vary regimen reported the highest scores (Table 3).

**Table 3:** The eight domains of LMQ

LMQ domain	Theoretical average of the domains*	Median IQR
Domain 1: Relationships with HCPs (5 items)	15.00	7(4)
Domain 2: Practical Difficulties in Using Medicines (7 items)	21.00	15(8)
Domain 3: Cost-Related Burden (3 items)	9.00	12(6)
Domain 4: Side Effects of Medicines (4 items)	12.00	10(9)
Domain 5: Effectiveness of prescribed medications (6 items)	18.00	10(7)
Domain 6: Concerns about Medicines Use (7 items)	21.00	24(9)
Domain 7 Impact of Using Medicines on Daily Life (6 items)	18.00	13(7)
Domain 8 Autonomy to Vary Regimen (3 items)	9.00	12(4)

HCPs, Healthcare Professionals; LMQ, Living with Medicines Questionnaire.

* Theoretical average of the domains: calculated if the answers to all questions were neutral

Univariate analysis revealed a significant association between the total LMQ score and male gender, suggesting that male patients might experience a higher medication-related burden. However, multivariate analysis found no patient-related variables independently correlated with the total LMQ score (Table 4).

**Table 4:** Correlation between LMQ and other variables in patients with UC

Variable	LMQ				
	Univariate		Multivariate		
	r	*P* value	Partial r	Standardized β	*P* value
**Age**	**-0.105**	**0.335**	**-**		**-**
**Sex**	**0.280**	**0.009 [S]**	**0.174**	**0.144**	**0.116**
**Disease duration**	**-0.154**	**0.158**	**-**	**-**	**-**
**Social**	**-0.006**	**0.959**	**-**	**-**	**-**
**Education**	**0.132**	**0.226**	**-**	**-**	**-**
**Governorate**	**0.128**	**0.239**	**-**	**-**	**-**
**Place of residence**	**0.055**	**0.617**	**-**	**-**	**-**
**Income**	**0.020**	**0.858**	**-**	**-**	**-**
**Biological type**	**0.136**	**0.210**	**-**	**-**	**-**
**Medication number**	**0.143**	**0.189**	**-**	**-**	**-**
**No. of comorbidities**	**0.100**	**0.360**	**-**	**-**	**-**

Linear regression analysis, r: regression coefficient

## DISCUSSION

Inflammatory bowel disease is a multifaceted condition that often necessitates the use of multiple drugs for symptom management and the maintenance of remission [23]. The chronic and recurrent characteristics of UC necessitate individuals to undergo either continuous or intermittent treatment throughout their lifetimes [4]. To our knowledge, this study was the first to measure MRB among patients living with UC. The current study indicated that 95.3% of patients with UC had varying degrees of MRB, with the majority (89.5%) having a minimum to moderate degree of MRB. This may be partially explained by the fact that 79.1% of the patients had no other comorbidities. Multimorbidity is linked with reduced quality of life, as well as higher hospitalization rates, healthcare expenses, resource demands, physiological distress, and complications from polypharmacy [24].

Moreover, the study identified that three domains registered the highest average scores, indicating pronounced concerns about cost-related burden, medicine use, and autonomy to vary regimens. This suggests that the participants experienced difficulties with medication costs, had high concerns about medicine use, and had limitations in changing the regimen. The high-cost burden reported is likely influenced by a notable proportion (68.6%) of the participants who obtained their medications through a combination of private sources and the Ministry of Health (MOH), with many depending on high-priced biological treatments. Inconsistencies in the availability of government-supplied medications throughout the year often necessitate patients purchasing drugs from private sources, exacerbating their financial strain. The IBD has a significant financial burden [25,26]. While traditional, less costly therapies were commonly used in the past, the advent of biologics has revolutionized the treatment of IBD but has also led to increased financial strain on healthcare systems [27]. The cost of biologics now surpasses other IBD-associated healthcare costs, including hospitalization and surgical procedures. A Dutch study highlighted that the cost of anti-TNF biologics considerably exceeds that of hospitalization, surgery, and even the loss of productivity for patients with CD and UC [28]. Another significant area of burden was the concerns about medicine use. Patient's opinions about their medication, or medication beliefs, include need and concern beliefs. The former includes the degree to which a person believes taking the drug is necessary to improve their health, while the latter includes worries or skepticism about medications [29].

Consequently, patients' medication beliefs can be classified into two primary domains: the perceived necessity of the medication for maintaining health and concerns about the possible negative implications of taking the medication, such as side effects or long-term dependency. There is a divergence of opinions among patients about the perceived efficacy of medications, with some asserting their positive impact. In contrast, others express concerns about the potential for adverse effects outweighing potential benefits [30]. Regarding the domain of autonomy to vary regimen, it is important to note that the medications used for treating UC, particularly biological drugs, were prescribed to all patients included in this study. These medications necessitate regular administration, which may explain the limitations of modifying the treatment regimen. Consequently, this restriction could contribute to the higher score observed in this domain.

Conversely, five domains displayed the lowest median burden scores. These included the quality of relationships with healthcare professionals (HCPs), the practicality of using medicines, concerns over side effects, the effectiveness of the medication, and the impact on daily living. Patients diagnosed with UC generally reported positive experiences regarding the efficacy of their medications, their interactions with healthcare providers, manageable use of their medicines, few concerns over side effects, and little disruption to their daily activities due to medication regimens. A previous study conducted in Iraq investigated the attitudes of patients with IBD towards medications, revealing that a significant proportion (58%) of participants expressed a high level of confidence in the efficacy of therapy [31]. The relationship between healthcare professionals and patients is evolving, significantly impacting treatment outcomes. The association between the burden of treatment and healthcare practitioners' inadequate provision of treatment-related information was identified. The presence of inadequate communication between HCPs and patients is likely correlated with polypharmacy, leading to an increased treatment burden [32].

The present study demonstrated that, when conducting multivariate analysis, none of the factors exhibited an independent correlation with LMQ. Prior research has indicated a correlation between increased treatment burden and specific demographics, such as younger and female patients [33-35].

### Limitations

It is important to acknowledge certain limitations present in this investigation. First, the research was conducted at a single institution in Baghdad, which may limit the generalizability of the findings. Furthermore, it is important to consider the potential influence of recall bias on the accuracy of self-reported responses; however, this limitation is inherent to such types of studies. Moreover, the sample size is quite limited. Future studies should include a larger sample size and multiple centers across different Iraqi regions to enhance the generalizability of the results. Despite these limitations, this study addresses a significant knowledge gap regarding the medication-related burden in patients with UC in developing countries. It adds valuable insights to the existing literature and establishes a foundation for subsequent comparative studies within developing nations and globally.

## CONCLUSION

In summary, a significant number of patients with UC enrolled in the study indicated different levels of burden associated with their medication. Many of these patients reported at least a moderate degree of medication-related burden. In addition, three domains reported the highest scores: cost-related burden, concerns about medicines use, and autonomy to vary regimens.

## Data Availability

Underlying data are available upon request from the corresponding author.

## Personal thanks

We want to thank all participants in this study.
